# The use of doxazosin before adrenalectomy for pheochromocytoma: is the duration related to intraoperative hemodynamics and postoperative complications?

**DOI:** 10.1007/s11255-020-02539-2

**Published:** 2020-07-03

**Authors:** Hao Kong, Nan Li, Jie Tian, Zhengqing Bao, Lu Liu, Kai Wu, Ying Gao, Bo Jin, Zheng Zhang, Dong Fang, Junqing Zhang, Liqun Zhou

**Affiliations:** 1 Department of Anaesthesiology and Critical Care Medicine, Peking University First Hospital, No. 8 Xishiku Street, Xicheng District, Beijing, 100034 China; 2 Department of Urology, Peking University First Hospital, No. 8 Xishiku Street, Xicheng District, Beijing, 100034 China; 3 Institute of Urology, Peking University, No. 8 Xishiku Street, Xicheng District, Beijing, 100034 China; 4 National Urological Cancer Center, No. 8 Xishiku Street, Xicheng District, Beijing, 100034 China; 5 Andrology Center, Peking University First Hospital, No. 8 Xishiku Street, Xicheng District, Beijing, 100034 China; 6 Department of Endocrinology, Peking University First Hospital, No. 8 Xishiku Street, Xicheng District, Beijing, 100034 China; 7 Department of Clinical Laboratory, Peking University First Hospital, No. 8 Xishiku Street, Xicheng District, Beijing, 100034 China

**Keywords:** Doxazosin (DOX), Pheochromocytoma, Duration, Hemodynamic, Hypotension, Bradycardia

## Abstract

**Purpose:**

No conclusion exists for the optimum duration of preoperative administration of doxazosin (DOX) before adrenalectomy for pheochromocytoma. The purpose of this study is to investigate whether perioperative hemodynamics and postoperative outcomes are related to the duration of DOX administration.

**Methods:**

In total, 132 patients managed preoperatively with single α-receptor blocker DOX were enrolled. All patients underwent adrenalectomy for pheochromocytoma in the Department of Urology, Peking University First Hospital, between January 2001 and July 2019. Patients were divided into three groups based on the duration of preoperative administration of DOX: group A (≤14 days), group B (15–30 days), and group C (>30 days). Patient and tumor characteristics, intraoperative hemodynamics, and postoperative outcomes were recorded and compared.

**Results:**

These patients included 57 men and 75 women, with an average age of 48 years. Clinical characteristics, preoperative hemodynamics, medicine management and surgical approaches were comparable between the three groups. Among the three groups, we found that group C (>30 days) had the lowest intraoperative minimum heart rate [group A vs. group B vs. group C = 60 (52–67) vs. 59 (50–61) vs. 51.5 (50–58.75), *p* = 0.024] and highest risk of postoperative hypotension requiring vasopressor support [group A vs. group B vs. group C = 14 (20.3%) vs. 12 (27.9%) vs. 10 (50.0%), *p* = 0.032].

**Conclusion:**

The current study indicated that preoperative management of pheochromocytoma with single α-receptor blocker DOX for more than 30 days after final dose adjustment might lead to intraoperative bradycardia and more postoperative hypotension requiring vasopressor support. Thus, our study does not support long-term (over 30 days) preoperative administration of pheochromocytoma with single α-receptor blocker DOX in the final dose.

## Introduction

Pheochromocytomas is a rare neuroendocrine vascular tumor with a low incidence of 0.8 per 100,000 people years [1]. Nearly 80–85% of pheochromocytomas arise from chromaffin cells in the adrenal medulla [2]. Because the release of catecholamine by pheochromocytomas differs among patients, a series of various clinical manifestations might present, typically including hypertension, diaphoresis, and tachycardia [3]. Currently, surgical resection is the only available curative treatment [4]. However, intraoperative tumor handling and accidental squeezing of the tumor can cause unpredictable and extensive catecholamine release into circulation [5]. This surgery leads to dangerous intraoperative hemodynamic instability changes, including rapid and severe blood pressure fluctuations that significantly increase the risk of major morbidity [6]. Preoperative hypotensive drugs could help decrease operative mortality and perioperative complications in patients undergoing pheochromocytoma removal from 45% to <1% and 69% to 3%, respectively [7, 8]. The Clinical Practice Guidelines of the Endocrine Society in the United States of America recommend that all patients with hormonally functional pheochromocytoma receive preoperative α-adrenergic receptor blockers for 7–14 days. However, no distinction is made between selective and nonselective α-blockers, and it is unclear whether selective α-blockers require more extended preoperative medication management.

It should be noted that the current duration of preoperative medicine administration is based on the clinical experience of doctors and not high-quality evidence-based research [9]. Although nonselective adrenergic blockers (phenoxybenzamine, PXB) are the most common protocol [10], retrospective studies demonstrated that α1-selective adrenergic receptor blockers (doxazosin, DOX) lead to fewer α2-driven side effects and could avoid prolonged postoperative hypotension [1]. Our previous study demonstrated no additional benefits of long-term (over 30 days) preoperative administration of PXB [11]. However, the optimal duration of preoperative use of DOX is debated. Therefore, this study aimed to investigate whether perioperative hemodynamics and postoperative outcomes are related to the duration of single α-receptor blocker DOX.

### Patients and methods

Data were reviewed from patients who underwent adrenalectomy for pheochromocytoma in the Department of Urology, Peking University First Hospital, between January 2001 and July 2019. A total of 174 patients received DOX before surgery and were initially considered for this study, 42 of whom were excluded, including 12 patients with bilateral or multiple pheochromocytomas, 21 patients with incomplete data, 7 patients with tumor recurrence and 2 patients with extra-adrenal tumors. Finally, after filtering, 132 patients were enrolled.

All patient medical records and imaging data were reviewed to collect information on patient demographics, blood pressure, heart rate (HR), surgical methods, tumor size, and tumor laterality. Preoperative maximum systolic and diastolic blood pressure (SBP and DBP) and HR were defined as the largest values obtained prior to initiation of surgical treatment. Additionally, we defined the SBP, DBP, and HR values 1 day before the operation as the preoperative SBP, DBP, and HR.

All patients received at least one catecholamine test before surgery, such as adrenaline, norepinephrine, dopamine for plasma or urinary, or vanillylmandelic acid. We have tested metanephrines for patients with suspected pheochromocytoma since 2016. Therefore, only 25 of 132 patients in this study underwent metanephrine testing. All patients’ biochemical test values were considered to be positive and beyond the upper limit of the normal range.

All patients received single selective α-blockade DOX for preoperative medical preparation. The initial dosage of DOX was 4 mg orally once daily. Clinicians can increase or decrease the dose of DOX based on the patient’s daily blood pressure fluctuations. Eventually, we stabilized the patient's blood pressure below 140/80 mmHg, and with the peripheral circulation changes, the limbs were warm to the touch. Patients can be slightly tolerant to the side effects of doxazosin, such as orthostatic hypotension and nasal stiffness [2]. After α-blockade DOX was achieved, a β-blocker was added for additional heart rate control when necessary. Other hypertensive drugs such as angiotensin-converting enzyme inhibitors (ACEIs), angiotensin receptor blockers (ARBs), or calcium channel blockers (CCBs) were also considered when required. All patients were prepared for surgery with 1–2 L intravenous saline infusion in the evening before surgery.

After preoperative management with single α-receptor blocker doxazosin, all patients underwent adrenalectomy under general anesthesia by experienced surgeons. All patients received an arterial line for blood pressure monitoring. Blood pressure data were collected from anesthesia records. The intraoperative hemodynamic instability measurements collected included (1) maximum and minimum SBP and DBP; (2) number of patients with high SBP (criteria included ≥160 mmHg, 160–179 mmHg, ≥180 mmHg, 180–199 mmHg, ≥ 200 mmHg, 200–219 mmHg, and ≥220 mmHg); (3) SBP > 130% of basic SBP; (4) SBP < 80 mmHg; (5) SBP < 70% of basic SBP; (6) maximum and minimum HR; (7) HR > 120 bpm; (8) mean arterial pressure (MAP) < 60 mmHg; (9) number of patients with SBP ≥ 200 and/or MAP < 60 mmHg; (10) ∆SBP = SBP_max_ − SBP_min_; (11) ∆DBP = DBP_max_ − DBP_min_; and (12) ∆HR = HR_max_ − HR_min_. Basal SBP was defined as the patient’s SBP 1 day before surgery.

The duration of preoperative management, referred to as the duration of the final dose (DFD), ranged from the time of final dose adjustment to surgery. According to the DFD, 132 patients were divided into three groups: group A (≤14 days), group B (15–30 days), and group C (>30 days). Patient and tumor characteristics, intraoperative hemodynamics, and postoperative outcomes were recorded and compared among the three groups.

The primary outcome was intraoperative hemodynamic instability, and the secondary endpoint was postoperative complications. Complications directly related to hemodynamic stability included new arrhythmia and postoperative hypotension and use of vasopressor, and those not directly related to hemodynamic stability included pulmonary complications, postoperative stroke, wound infection, intestinal obstruction, urinary tract infection, acute liver injury after the operation, postoperative acute kidney injury, venous thrombosis, and hypoglycemia. The criteria of the Japan Clinical Oncology Group were used to evaluate the severity of the complications [12].

### Statistical analysis

Statistical analysis was performed using SPSS version 20.0 (IBM Corp, Armonk, NY, USA), and statistical significance was set at *p* < 0.05. Pearson’s test and the chi-square test were used to test the distribution of categorical variables, and the *F* test was applied for continuous variables. The Kruskal–Wallis test was used for non-normally distributed variables.

## Results

### Patient and tumor characteristics

A total of 132 patients were enrolled in this study, including 57 men and 75 women, with an average age of 48 years. All patients were divided into three groups: 69 cases in group A (≤14 days), 43 cases in group B (15–30 days) and 20 cases in group C (>30 days). The age, sex, body mass index (BMI), tumor laterality, tumor size, biochemically positive status and perioperative drug management in the three groups were comparable, as shown in Table 1. We found no significant difference in preoperative hemodynamics.

**Table 1:** Patient and tumor characteristics

	Group A	Group B	Group C	Overall	*p*
No. of cases, *n*	69	43	20	132	
Age	46.41 ± 13.86	49.35 ± 15.16	52.50 ± 15.55	48.29 ± 14.61	0.221
Gender					0.162
Female	34 (49.3%)	27 (62.8%)	14 (70.0%)	75 (56.8%)	
Male	35 (50.7%)	16 (37.2%)	6 (30.0%)	57 (43.2%)	
BMI	23.95 (20.98–26.14)	23.05 (21.30–25.10)	22.88 (20.25–24.22)	23.05 (21.15–25.48)	0.470
Preoperative BP					
SBP	125 (115–130)	124 (112–132)	119 (111–144)	124 (112.25–131)	0.951
DBP	78 (70–81)	72 (68–80)	72.5 (67–77)	75 (69–80)	0.063
Preoperative HR	76 (72–79)	76 (72–80)	78 (73–80)	76 (72–80)	0.746
Preoperative BP_max_					
SBP_max_	160 (136–181)	160 (130–200)	180 (151–189)	160 (135–190)	0.604
DBP_max_	94 (80–110)	90 (77–120)	92 (86–108)	92 (80–110)	0.954
Surgical approach					0.519
Open	11 (15.9%)	10 (23.3%)	5 (25.0%)	26 (19.7%)	
Endoscopic	58 (84.1%)	33 (76.7%)	15 (75.0%)	106 (80.3%)	
Size of tumor, cm	5.00 (4.00–6.55)	5.10 (3.60–6.20)	4.90 (4.13–6.43)	5.00 (4.00–6.50)	0.972
Tumor laterality					0.179
Left	23 (33.3%)	21 (48.8%)	10 (50.0%)	54 (40.9%)	
Right	46 (66.7%)	22 (51.2%)	10 (50.0%)	78 (59.1%)	
Biochemical positive	50 (72.5%)	34 (79.1%)	16 (80.0%)	100 (75.8%)	0.649
Average dose of DOX	4 (4–4)	4 (4–4)	4 (4–8)	4 (4–4)	0.674
Cases of β-blockers	10 (14.5%)	12 (27.9%)	5 (25.0%)	27 (20.5%)	0.198
Cases of ACEIs	6 (8.7%)	3 (7.0%)	0 (0.0%)	9 (6.8%)	0.565
Cases of ARBs	5 (7.2%)	7 (16.3%)	2 (10.0%)	14 (10.6%)	0.339
Cases of CCBs	17 (24.6%)	15 (34.9%)	5 (25.0%)	37 (28.0%)	0.476

*BMI* body mass index, *BP* blood pressure, *SBP* systolic blood pressure, *DBP* diastolic blood pressure, *HR* heart rate, *DOX* Doxazosin (mg/day), *ACEIs* angiotensin-converting enzyme inhibitors, *ARBs* angiotensin receptor blockers, *CCBs* calcium channel blockers

### Intraoperative hemodynamics and postoperative outcomes

The detailed data of intraoperative hemodynamics are shown in Table 2. Among the three groups, we found no significant difference in intraoperative minimum SBP (*p* = 0.260) or DBP (*p* = 0.300), intraoperative maximum SBP (*p* = 0.222) or DBP (*p* = 0.091), intraoperative maximum HR (*p* = 0.756), ∆SBP (*p* = 0.519), ∆DBP (*p* = 0.591) or ∆HR (*p* = 0.339). No significant difference was noted for the incidence of intraoperative minimum SBP < 80 mmHg (*p* = 0.271), intraoperative maximum SBP ≥ 160 mmHg (0.306), intraoperative maximum SBP ≥ 220 mmHg (0.666), intraoperative minimum HR (*p* = 0.756), MAP < 60 mmHg (*p* = 0.534), SBP < 70% of basic SBP (*p* = 0.405), SBP > 130% of basic SBP (*p* = 0.665), SBP ≥ 200 mmHg or MAP < 60 mmHg (*p* = 0.904). We further stratified data on the basis of blood pressure values but did not find a statistically significant difference in the number of people with SBP 160–179 mmHg (*p* = 0.579), the number of people with SBP 180–199 mmHg (*p* = 0.919), the number of people with SBP 200–219 mmHg (*p* = 0.565), intraoperative maximum SBP ≥ 180 mmHg (*p* = 0.559), or intraoperative maximum SBP ≥ 200 mmHg (*p* = 0.483). However, we found that group C (>30 days) had a lower intraoperative minimum heart rate (HR) [group A vs. group B vs. group C = 60 (52–67) vs. 59 (50–61) vs. 51.5 (50–58.75), *p* = 0.024].

**Table 2:** Intraoperative hemodynamics of the three groups

	Group A	Group B	Group C	Overall	*p*
Intraoperative SBP_min_	90 (82–100)	96 (90–102)	90 (76–103)	90 (84–100)	0.260
Intraoperative DBP_min_	50 (46–60)	55 (48–60)	50 (43–59)	50 (46–60)	0.300
SBP < 80 mmHg, *n*	9 (13.0%)	4 (9.3%)	5 (25.0%)	18 (13.6%)	0.271
SBP ≥ 160 mmHg, *n*	45 (65.2%)	30 (69.8%)	10 (50.0%)	85 (64.4%)	0.306
160 mmHg ≤ SBP < 180 mmHg, *n*	22 (31.9%)	12 (27.9%)	4 (20.0%)	38 (28.8%)	0.579
SBP ≥ 180 mmHg, *n*	23 (33.3%)	18 (41.9%)	6 (30.0%)	47 (35.6%)	0.559
180 mmHg ≤ BP < 200 mmHg, *n*	13 (18.8%)	8 (18.6%)	3 (15.0%)	24 (18.2%)	0.919
SBP ≥ 200 mmHg, *n*	10 (14.5%)	10 (23.3%)	3 (15.0%)	23 (17.4%)	0.483
200 mmHg ≤ SBP < 220 mmHg, *n*	6 (8.7%)	6 (14.0%)	1 (5.0%)	13 (9.8%)	0.565
SBP ≥ 220 mmHg, *n*	4 (5.8%)	4 (9.3%)	2 (10.0%)	10 (7.6%)	0.666
< 70% of basic SBP	24 (34.8%)	12 (27.9%)	9 (45.0%)	45 (34.1%)	0.405
> 130% of basic SBP	39 (56.5%)	28 (65.1%)	12 (60.0%)	79 (59.8%)	0.665
Intraoperative SBP_max_	170 (149–183)	170 (150–198)	159 (145–180)	170 (150–188)	0.222
Intraoperative DBP_max_	90 (80–105)	99 (90–102)	89 (76–100)	91 (84–101.5)	0.091
Intraoperative HR_max_	90 (80–101.5)	91 (80–100)	86 (71–108)	90 (79–101)	0.756
Intraoperative HR_min_	60 (52–67)	59 (50–61)	52 (50–59)	59 (50–61)	0.024^*^
MAP < 60	17 (24.6%)	7 (16.3%)	5 (25.0%)	29 (22.0%)	0.534
∆SBP	71 (55–92)	79 (60–105)	75 (50–101)	75 (57–95)	0.519
∆DBP	40 (30–50)	40 (31–57)	39 (27–53.5)	40 (30–53)	0.591
∆HR	30 (20–40)	34 (24–46)	34 (20–48)	31 (20–44)	0.339
SBP ≥ 200 mmHg and/or MAP < 60 mmHg	24 (34.8%)	16 (37.2%)	8 (40.0%)	48 (36.4%)	0.904

∆SBP = SBP_max_ – SBPmin; ∆DBP = DBP_max_ – DBP_min_; ∆HR = HR_max_ – HR_min_

*SBP*_*min*_, minimum systolic blood pressure, *SBP*_*max*_ maximum systolic blood pressure, *DBP*_*min*_ minimum diastolic blood pressure, *DBP*_*max*_ maximum diastolic diastolic blood pressure, *HR*_*max*_ maximum heart rate, *MAP* mean arterial pressure

*Statistically significant

### Complications

Finally, we found 47 people with 76 complications in our research. Except for one patient who died postoperatively, the remaining patients recovered well and were discharged after symptomatic treatment. These complications are summarized in Table 3.

**Table 3:** Summary of postoperative complications base on the relationship with hemodynamics

Variable	All complications *n* = 76
Directly related to hemodynamic stability	
New arrhythmia	3
Postoperative hypotension and use of vasopressor	36
Not directly related to hemodynamic stability	
Pulmonary complications	5
Postoperative bleeding	2
Postoperative intestinal obstruction	1
Acute liver injury after the operation	7
Postoperative acute kidney injury	6
Venous thrombosis	2
Hypoglycemia	2
Death	1

As shown in Table 4, according to the requirement of the intensive care unit (ICU), the days in the ICU, complications, and postoperative hospitalization exhibited no significant difference (*p* = 0.218, 0.327, 0.264, and 0.633, respectively).

**Table 4:** The major postoperative outcome of the three groups

	Group A	Group B	Group C	Overall	*p*
Cases of ICU needed, *n*	42 (60.9%)	25 (58.1%)	16 (80.0%)	83 (62.9%)	0.218
ICU, day	1 (0–1)	1 (0–1)	1 (1–1)	1 (0–1)	0.327
Postoperative hypotension and vasopressor support, *n*	14 (20.3%)	12 (27.9%)	10 (50.0%)	36 (27.3%)	0.0328^*^
Complications, *n*	21 (30.4%)	16 (37.2%)	10 (50.0%)	47 (35.6%)	0.264
Postoperative hospitalization, day	5.0 (3.5–7.0)	5.0 (4.0–7.0)	5.0 (3.3–7.0)	5.0 (4.0–7.0)	0.633

*ICU* intensive care unit

*Statistically significant

However, we found that group C (>30 days) had the greatest incidence of postoperative hypotension requiring vasopressor support [group A vs. group B vs. group C = 14 (20.3%) vs. 12 (27.9%) vs. 10 (50.0%), *p* = 0.032] (Table 4).

## Discussion

The history of preoperative medical preparation in patients with pheochromocytoma encompasses nearly 70 years [6]. Preoperative α-adrenergic receptor blockers in patients with pheochromocytoma can not only normalize blood pressure and heart rate but also reverse catecholamine-induced blood volume contraction preoperatively [13]. Even preoperative management has been proven to reduce the risk of intraoperative hemodynamic instability and postoperative complications [3, 6, 14].

Since 1988, doxazosin, a selective α-antagonist, has been widely adopted in the preoperative management of patients with pheochromocytoma [15]. Compared with nonselective α receptor blockers, DOX can reduce the incidence of tachycardia caused by blockage of the α2-adrenoceptor on the presynaptic membrane that affects norepinephrine uptake or release [16]. DOX was associated with fewer sustained postoperative hypotension events and better postoperative hemodynamic recovery than were nonselective adrenergic blockers [13]. The duration of preoperative administration of DOX was negatively correlated with the maximum intraoperative blood pressure [17]. However, the optimal duration of preoperative α1-selective adrenergic receptor blockers remains unclear [3], and evidence for the most appropriate duration of preoperative preparation with DOX is lacking. Currently, clinicians often rely on experience and their own institution to determine the time of preoperative medication management. Certain researchers have reported a median duration of DOX of more than 4 weeks [5, 10, 18]. The median duration of preoperative management with DOX in our center is 15 days. Different from previous studies on the definition of time of preoperative management, our research defined the duration as the time between final dose adjustment and surgery.

Our result indicated that preoperative management of pheochromocytoma with single α-receptor blocker DOX more than 1 month after the final dose adjustment could lead to intraoperative bradycardia, which was not reported in previous studies. Two studies showed that the duration of preoperative management does not influence intraoperative hemodynamic fluctuations and postoperative complications. Russell [19] et al. found that the duration of preoperative management is not related to the stability of surgery. However, the sample size in that study was only 14 people, the evaluation index for stable operation was simple, and the longest medication time was no more than 15 days. Hack et al. reached a similar conclusion, but the study was limited in focusing only on young patients and having a small sample size [20]. The effect on HR was not reported in these studies. Although the α1-blocker has a limited impact on HR theoretically, our study demonstrated that long-term administration might significantly reduce HR and might increase intraoperative risk. However, the reason why prolonging the duration of preoperative treatment with the α1-selective adrenoceptor blocker doxazosin (DOX) will affect the heart rate of surgery needs further research.

Our results also show that patients with DOX preoperative medication for more than 1 month after the final dose adjustment have a greater incidence of postoperative hypotension requiring vasopressor support. Reese [5] et al. demonstrated that DOX causes more cases of SBP < 80 than PXB in 52 patients with pheochromocytoma undergoing laparoscopic surgery. Furthermore, postoperatively, the DOX group requires more additional support with vasopressor infusions under PACU or ICU admission. However, no significant differences in postoperative discharge time were observed between the 3 groups. It should be noted that compared with that from PXB data [11], the proportion of postoperative hypotension was relatively low, which was generally in accordance with the findings of previous reports on the difference between DOX and PXB. Nevertheless, long-term administration of DOX could also relatively increase this risk.

In our study, the clinical characteristics, catecholamine test results, and surgical approaches were comparable among the three groups, and we found no significant differences in preoperative hemodynamics or medication management. All of these observations make our conclusion more reliable, proving that a longer duration of DOX not only confers no benefit in terms of perioperative hemodynamics but also might lead to a higher risk of postoperative hemodynamic stability-related complications.

There are certain limitations to this study. First, this study represents a retrospective review of data at a single center, which might incur selective and recall bias, and further external validation (especially in non-Chinese cohorts) is necessary. Second, since 2016, we have performed metanephrine testing for patients with suspected pheochromocytoma. Therefore, of the 132 patients in this group, only 25 underwent metanephrine testing. Although all patients were positive in the metanephrine test, this index could not be included in the risk factor analysis. Finally, a large sample of prospective randomized controlled studies is needed to further verify the conclusion.

## Conclusion

The current study indicated that the preoperative management of pheochromocytoma with single α-receptor blocker DOX for more than 30 days after final dose adjustment might lead to intraoperative bradycardia and a heightened incidence of postoperative hypotension requiring vasopressor support. Thus, our study does not support long-term (over 30 days) preoperative administration of pheochromocytoma with single α-receptor blocker DOX in the final dose.
